# Response to Letter to the Editor: Delayed Presentation of Non-COVID-19 Patients During the COVID-19 Pandemic Is Not Limited to Children

**DOI:** 10.5041/RMMJ.10448

**Published:** 2021-07-20

**Authors:** Yonatan Yeshayahu

**Affiliations:** Pediatrics Department, Samson Assuta Ashdod University Hospital, Goldman Medical School, Ben-Gurion University, Ashdod, Israel

**Keywords:** Children, COVID-19, delayed diagnosis

## TO THE EDITOR

I read with interest the letter by Klaus Rose et al. regarding the article about delayed diagnosis of severe medical conditions during the coronavirus disease 2019 (COVID-19) pandemic.^1^ The authors stressed the importance of recognizing that delayed presentation of patients, during the COVID-19 pandemic, was not limited to the pediatric population. I agree with this important point, and the article does not claim otherwise. The presented cases are pediatric, given that they took place in a pediatric department, but it is reasonable to assume that adults have faced the same challenges.

The authors raise a question of the need to differentiate between adolescents and adults given that some of them are physiologically like adults, and that the differentiation is for administrative reasons. This seems a technical differentiation, but it is not. Specifically in this article, we deal, not with a medical condition unique to pediatrics, but rather with parental decisions regarding their children. This is where the administrative reasons and the medical reasons meet. A 16-year-old, even if physiologically he resembles an adult, still cannot decide for himself to go the emergency room, and cannot be admitted to the hospital or treated without parental approval. Adults can make decisions whether to seek medical treatment, and deal with the consequences of their decisions. Adolescents have their parents make decisions on their behalf, and therefore they need to receive special consideration, and protection. In the case of the 16-year-old, as mentioned in the article, the parents received medical recommendation for admission and treatment, but opted to go home. Their decision had a long-term impact on their child, which is different than an adult making this decision for himself and facing the consequences. For this reason, I think that special consideration should be given to the pediatric population, just like we require parental consent for participating in research, even in a 17-year-and-11-month-old adolescent.

Nevertheless, the message regarding delayed diagnosis during the COVID-19 pandemic, leading to medical complications, is true for all the population, adults and children.

## Abbreviations

COVID-19: coronavirus disease 2019
